# Supplementary material for: Are carbon emissions trading and green financial instruments synergistic? -Comprehensive quantitative research based on content analysis
Source: PLoS One. 2024 Mar 7;19(3):e0298601. doi: 10.1371/journal.pone.0298601 (PMC10919601; doi:10.1371/journal.pone.0298601)
Supplement: S1 Data — (ZIP) [file pone.0298601.s001.zip › Data/地级市Z_F工作报告.zip!/╔╜╢1⁄2/┴─│╟╩╨/└┤╫╘╣1⁄2╓┌║┼í╛┬φ┐╦╩2╛▌═°í┐_2017.docx]

各位代表：

　　现在，我代表市人民政府向大会报告工作，请予审议，并请市政协各位委员提出意见。

　　一、2016年工作回顾

　　2016年是我市在复杂环境下继续砥砺前行、取得新的突破的一年。一年来，在省委省政府和市委的坚强领导下，全市上下按照“作风引领、团结实干、争先进位、科学发展”的总体要求，改革创新，攻坚克难，市第十六届人大六次会议确定的主要目标任务顺利完成，实现了“十三五”良好开局。

　　(一)综合经济实力实现新提升。全市经济保持平稳健康发展，主要指标增幅居全省前列。全年生产总值2859.2亿元，比上年增长7.3%;地方一般公共预算收入187.5亿元，同口径增长8.6%，总量在全省前进了两个位次;固定资产投资2357.6亿元，增长12.2%;社会消费品零售总额1173.1亿元，增长10.7%;本外币各项存贷款余额分别达到3032.5亿元和2130.4亿元，较年初增长17%和13.8%;城乡居民人均可支配收入分别达到23277元和11387元，增长7.9%和8.3%。

　　(二)产业转型升级迈出新步伐。“三去一降一补”取得明显进展，经济发展质量进一步提高。农业供给质量稳步提升。粮食总产达到124亿斤，实现“十四连丰”;瓜菜菌总产1702万吨，肉蛋奶总产197万吨，均保持全省前列;新增规模以上龙头企业27家、专业合作社1379家、家庭农场308家;新增“三品一标”210个，其中绿色食品174个，连续三年位居全国地级市首位。成功推出“聊·胜一筹”农产品区域公用品牌，叫响了“放心吃吧，聊城产的”宣传口号。农田水利基础设施建设三年会战全面完成，治理沟渠5142条，改善除涝面积570万亩，南水北调配套工程七个水库基本建成。农作物耕种收综合机械化率达到87%。工业提档升级进一步加快。“双百工程”完成投资462亿元，其中投资84亿元的信源高纯铝、投资34亿元的鲁西集团聚碳酸酯等25个项目建成投产;新增主营业务收入过百亿元企业3家。信发高新材料产业园10个入园项目总投资97亿元。全市新增山东省名牌产品8个、著名商标20件，中通客车荣获省长质量奖。服务业扩量提质扎实推进。完成了《京杭运河(聊城段)旅游发展及水生态体系规划》，组建了聊城旅游投资发展集团，水上古城成为中国自驾游首选目的地，阿胶景区入选首批国家工业旅游创新单位，东阿县获批创建国家全域旅游示范区，全市旅游消费总额增长13.5%。电子商务交易额达到935.8亿元，增长43%。全市服务业增加值增长9.6%，增幅居全省第4位，服务业占比提高了1.6个百分点。

　　(三)新旧动能转换取得新成效。坚持以创新增动力。新增国家级众创空间4家、省级创新平台18家，莘县农业科技园获批创建国家级园区，东阿阿胶“国家胶类中药工程技术研究中心”通过验收，国家级铜铝冶炼及加工产品质检中心已经建成，聊城大学质量学院正式成立并已招生，高新技术产业占比提高了1.9个百分点。大力推进“大众创业、万众创新”，新增省级创新孵化基地1家、博士后创新实践基地4家，市创业大学被认定为全省首批示范创业大学;全市新登记市场主体5.6万户、私营企业1.7万家，分别增长13.4%和31.3%。坚持以改革激活力。农村土地承包经营权确权登记颁证工作以优秀等次通过验收，新型农村合作金融试点实现全覆盖，国有林场改革在全省率先启动。新成立了财金公司、土储集团;设立了注册资金10.2亿元的市级担保公司，资本规模居全省前列。推出了国内首支市级股权质押增信基金、省内第三支市级直投基金，新增“新三板”挂牌企业10家，列全省第6位;新增齐鲁股权交易市场挂牌企业263家，列全省第2位。坚持以开放促发展。全市招商引资到位资金803.9亿元，引进外国专家160名。完成进出口总额375.4亿元，增长19.5%，其中出口197.8亿元，增长25.4%，增幅居全省第1位;实际利用外资4.4亿元，增长26.8%，增幅居全省第2位;对外投资20.7亿元，增长104%，增幅居全省第4位。过去的一年，全市科技创新步伐进一步加快，改革开放向纵深推进，新旧动能加快接续转换，推动实体经济发展的新动力正在加速形成。

　　(四)新型城镇化建设开创新局面。规划体系更加健全。完成了聊城市城市总体规划、新型城镇化规划等综合性规划，以及城市地下综合管廊、海绵城市、绿地系统、城区水系和水上公交等专项规划。城市功能更加完善。全市新建改建城市道路192.6公里，建成污水处理厂4家;开工建设综合管廊6.9公里，建成4.2公里。中心城区实施了供暖“汽改水”工程，新增供暖面积244万平方米;新增纯电动公交车220辆，实现节能环保车全覆盖;建设了公共自行车出行系统，投放自行车1万辆。环境整治更加深入。美丽乡村规范化建设全面启动，城乡环卫一体化、路域环境整治、小城镇提升等深入实施;农村改厕工程完成27.6万户，荣获山东省人居环境范例奖。基础支撑更加有力。德上高速聊城至夏津段建成通车，青兰高速、莘南高速加快建设，国省道改建大中修工程完成197公里，新改建农村公路1996公里，京九高铁、郑济高铁、聊城机场、聊泰铁路、德单高速等项目前期工作扎实推进。各位代表，2016年，我市不仅城乡面貌发生了新的变化，而且成功列入中原城市群发展规划，被确定为国家新型城镇化综合试点、山东省百万人口大城市试点和首批海绵城市试点，机遇在叠加，建设在加速，环境在提升，特色在彰显，聊城正在向现代化区域中心城市阔步迈进!

　　(五)生态环境质量获得新改善。落后产能淘汰扎实推进。全市淘汰小火电机组10台、114万千瓦，10万千瓦及以上机组均完成超低排放改造。压减钢铁产能70万吨，关停“地条钢”企业12家，关停了鲁西集团一厂、二厂、四厂和昌润金热电。整顿化工企业206家，关停153家。大气污染治理持续加力。开展了“百日攻坚”集中行动，取缔了禁燃区内所有非集中供热燃煤锅炉，建设清洁煤配送网点112个，全市细颗粒物、可吸入颗粒物、二氧化硫、二氧化氮浓度分别改善14.9%、7.9%、24.4%和6.8%，“蓝天白云”天数增加29天。水污染防治成效明显。周公河湿地一期土建工程已经完工，“千亩表流、百亩潜流”人工湿地实现县(市)全覆盖，代表山东省参加海河流域水污染核查再次荣获第一名。生态绿化步伐加快。积极创建国家森林城市，启动了8个国有林场提升改造，完成植树造林20.8万亩，全市林木绿化率达到40%。

　　(六)群众生活质量有了新提高。脱贫攻坚成果显著。大力实施千企下乡、万户养驴等特色扶贫工程，全市实施扶贫项目576个，完成投资47.5亿元，共退出贫困村253个，脱贫16.9万人。民生保障进一步加强。全市新增城镇就业5.3万人，登记失业率控制在3.09%，社保基金征缴总额增长12.2%。新增养老床位3932张，新建农村幸福院121处。棚户区改造新开工建设2.05万套，老旧小区改造完成1.6万户，农村危房改造完成8225户，均超额完成年度任务。社会事业全面发展。11所城市公立医院综合改革全面启动，东昌府、临清、东阿开展了分级诊疗试点，全省疾控系统首家院士工作站落户我市;全面两孩政策平稳实施，卫生计生“五项免费”工程惠及群众29.2万人。中小学校长职级制和去行政化改革全面推开，153所市区学校兼并托管了212所农村学校;“大班额”问题综合完成率63.6%，“全面改薄”竣工率87.2%，均超额完成省定任务。建成基层综合文化服务中心3936个，开展文化惠民公益演出2975场，水城明珠剧场改造全面完工，电视无线数字覆盖工程建成并投入使用。全民健身运动蓬勃发展，我市运动员在全国以上比赛中荣获金牌4枚。社会保持和谐稳定。平安聊城建设扎实推进，安全生产事故起数和死亡人数全面下降，食品药品市场秩序进一步规范。实施了“一乡镇一团队、一村居一顾问”工程，全市信访总量下降16.9%。全国双拥模范城创建实现“四连冠”。新闻出版、社会科学、档案史志、民族宗教、外事侨务、台湾事务、气象地震、妇女儿童、青少年、残疾人、老龄、人防、油区、对口支援等工作，都取得新的成绩。

　　(七)政府自身建设得到新加强。深入贯彻党的十八届六中全会精神，认真开展“两学一做”学习教育。配合市人大常委会制定地方性法规和决定决议9件。严格执行人大及其常委会的决定决议，主动加强与政协的民主协商，办理人大代表议案建议163件、政协委员提案204件，满意率和基本满意率均达到99%以上。积极倡导“认真、专业、创新、高效、务实”的工作作风，深入落实“星期六企业工作日”、“星期六群众工作日”和“金融工作日”制度，帮助企业解决问题274个，化解重点信访案件92件。党政机关公务用车制度改革如期完成，事业单位分类改革有序推进。削减市级行政审批事项46项，“互联网+政务服务”走在全省前列。实行了企业登记“五证合一”、“一照一码”，市公共资源交易中心投入运行。市长公开电话在全国第三方机构313条热线监测中，荣获并列第1名。正风肃纪持续发力，全市纪检监察机关立案查处违纪问题2337件，党纪政纪处分2649人。

　　各位代表，2016年是本届政府工作的收官之年。经过五年的努力，我市经济社会发展取得了巨大成就。区域地位显著提升。我市先后被列入中原经济区、中原城市群、京津冀协同发展区、省会城市群经济圈和山东西部经济隆起带，成为国家和省区域战略叠加区;同时获批为国家现代农业示范区、国家农业科技园区、国家农业可持续发展试验示范区、国家节能减排财政政策综合示范城市、国家质量强市示范城市、国家循环经济示范城市、国家新型城镇化综合试点地区，在多个领域正在跨入国内领先行列。综合实力显著增强。2016年，全市生产总值是2011年的1.6倍，年均增长9.6%，高于全省0.9个百分点;地方一般公共预算收入是2011年的1.9倍，年均增长14.2%，高于全省3.1个百分点;本外币存贷款余额分别比2011年增加1666亿元和1019.7亿元。产业素质显著提高。粮食生产达到“吨粮市”标准，我市被评为“中国蔬菜第一市”，“三品一标”数量是2011年的7.1倍。规模以上工业增加值是2011年的1.8倍，高新技术产业占比提高了13.6个百分点。服务业占生产总值比重提高了8.5个百分点。城乡面貌显著变化。市县城区建成区面积由210平方公里增加到300平方公里，城市人口由223.3万人增加到292.8万人，常住和户籍人口城镇化率分别提高了10.3和5.1个百分点;城市绿化面积由8516公顷增加到1.3万公顷。全市新增高速公路164公里、一级公路73公里，新建改建农村公路6765公里，小城镇建设完成投资127亿元，建成农村新型社区510个。群众生活显著改善。全市累计新增城镇就业32.2万人、农村劳动力转移就业38.7万人，基本社会保险覆盖率达到87.5%。城乡居民人均可支配收入分别是2011年的1.6倍和1.7倍。

　　各位代表，过去五年，我市经济社会发展取得的成绩，是中央、省委省政府和市委坚强领导的结果，是全市上下团结奋斗的结果，是各级各方面大力支持的结果。在此，我代表市人民政府，向全市人民，向人大代表、政协委员，向民主党派、工商联、人民团体和各界朋友，向驻聊部队官兵和中央、省驻聊各单位，向所有关心支持聊城发展的海内外朋友，致以崇高的敬意和衷心的感谢!

　　各位代表，在看到成绩的同时，我们也清醒地认识到我市在发展中还存在不少困难和问题：经济发展速度还不够快，经济总量和人均占有水平依然不高;经济结构不尽合理，服务业发展滞后，重化工业比重较高;城镇化率相对较低，城镇规划建设管理水平有待提升;生态治理压力依然较大，重污染天数仍然较多;民生事业仍有不少短板，公共服务设施还不完善;政府职能转变还不到位，发展环境还不够优化;干部队伍存在不担当不作为慢作为现象，廉政建设和反腐败斗争的任务依然艰巨。对此，我们一定高度重视，强化责任担当，积极予以解决。

　　二、今后五年的目标任务

　　各位代表，今后五年是我市全面深化改革的攻坚期、全面建成小康社会的决胜期。根据市十三次党代会精神，今后五年政府工作的总体要求是：全面贯彻党的十八大和十八届三中、四中、五中、六中全会精神，深入落实习近平总书记系列重要讲话精神，统筹推进“五位一体”总体布局，协调推进“四个全面”战略布局，积极践行五大发展理念，坚持作风引领、团结实干、争先进位、科学发展，坚持“一二三四五一”的总体思路，努力形成经济发展、政治清明、文化繁荣、社会和谐、生态良好的新格局，确保与全省同步全面建成小康社会，奋力谱写聊城人民幸福生活新篇章。主要预期目标是：生产总值和地方一般公共预算收入年均增长8%左右，固定资产投资年均增长13%，社会消费品零售总额年均增长10%，城乡居民人均可支配收入年均分别增长8%和9%左右。

　　(一)更加突出创新引领，实现质量效益大提升。大力实施大项目带动、创新驱动、质量强市战略，深化供给侧结构性改革，加快经济转型升级。推进农业现代化，建成国家现代农业示范区。推进传统产业改造和战略新兴产业培育，全面建成国家节能减排财政政策综合示范城市、国家循环经济示范城市、国家质量强市示范城市，高新技术产业占比达到35%以上。推进服务业发展提速、质态提升，服务业占比达到47%以上。

　　(二)更加突出统筹协调，实现城乡面貌大变化。加快推进以人为核心的新型城镇化，构建城乡协调发展新格局。抓好“聊茌东”都市区建设，强化中心城区龙头作用，推动茌平、东阿撤县设区，促进组团发展、紧凑布局、精明增长，城镇人口达到200万人以上。抓好县(市)城区建设，积极发展小城镇，稳妥推进新型农村社区建设，全市常住和户籍人口城镇化率分别达到56%和45%以上。

　　(三)更加突出绿色生态，实现环境质量大改善。加强生态文明建设，打造美丽宜居新家园。做好水的文章，抓好京杭运河聊城段保护开发，推进“以河代库、以堤代路、沿岸绿化、蓄水造景”，实现河湖相连、水系相通，打造全域水城。做好绿的文章，全面抓好城区、水系、道路、村镇、农田林网绿化，扩大绿化规模，提升绿化水平，建成国家森林城市。做好洁的文章，以治理大气污染为重点，严格工业项目准入，优化能源结构，加强资源集约节约利用，走出一条生产发展、生活富裕、生态良好的文明发展之路。

　　(四)更加突出协同融合，实现对外开放大突破。全面扩大对内对外开放，构建富有生机活力的开放型经济体系。加快区域融合发展，积极融入京津冀协同发展和国家中原经济区战略，全方位参与省会城市群经济圈和山东西部经济隆起带建设。推进各领域对外合作，努力扩大进出口，安全高效地建设“海外聊城”。大力开展招商引资、招才引智，推动市经济技术开发区进一步提升在“国家队”中的位次排名，促进市高新技术产业开发区建成国家级高新区、江北水城旅游度假区创建国家级旅游度假区。抓好京九高铁、郑济高铁、聊城机场等重大基础设施项目建设，构建立体化交通体系，尽早建成冀鲁豫三省交界综合交通枢纽城市。

　　(五)更加突出共享发展，实现群众生活大提高。坚持共建共享，切实实现好、维护好、发展好人民群众的根本利益。坚决打赢脱贫攻坚战，确保2018年圆满完成任务。实施更加积极的就业政策，健全水平更高、覆盖更广的社会保障体系，每年办成一批民生实事。积极发展各项社会事业，全面加强法治聊城、信用聊城、平安聊城、健康聊城建设，创建全国文明城市。

　　各位代表，经过五年的不懈奋斗，我市将实现综合实力大幅提升、内外交通快速便捷、城乡环境清新优美、群众生活幸福安康，一个经济繁荣、富有活力、秀美宜居、文明和谐的新聊城，将在齐鲁大地蓬勃崛起!

　　三、2017年工作重点

　　2017年是实施“十三五”规划的重要一年，是新一届政府工作的开局之年。我们要按照中央、省委省政府和市委的决策部署，坚持稳中求进工作总基调，牢固树立和贯彻落实新发展理念，以提高质量和效益为中心，以推进供给侧结构性改革为主线，统筹抓好稳增长、促改革、调结构、惠民生、防风险各项工作，确保全市经济平稳健康发展和社会和谐稳定。综合考虑多方面因素，建议全市经济社会发展主要预期目标为：生产总值增长7.5%左右，地方一般公共预算收入增长7%左右，固定资产投资增长12%左右，社会消费品零售总额增长10%左右，实际利用外资增长10%左右，进出口总额保持稳定增长，城乡居民人均可支配收入分别增长8%和8.5%左右，居民消费价格涨幅控制在3%左右，全面完成省下达的各类约束性指标。工作中，重点抓好以下七个方面：

　　(一)坚定不移抓好实体经济发展，全力推动产业转型升级。深入推进“三去一降一补”，努力扩大高质量产品和服务供给。大力推进农业供给侧结构性改革。一是强化产业化带动。着力推动“新六产”发展，促进农业与工业、旅游、文化深度融合，提高农民在产业融合发展中的收益。加大农业龙头企业培育力度，重点支持农产品加工，新增规模以上龙头企业30家。实施“一乡一业”、“一村一品”示范工程，抓好肉驴养殖、特种水产养殖基地建设，打造10个示范乡(镇)、200个示范村(居)。实施“净菜进京入沪”工程，争取京、沪商委在我市认定10处高端蔬菜生产基地，直供蔬菜总量力争翻番。二是强化组织化带动。探索农村土地“三权”分置实现形式，土地流转面积占比力争达到1/3。积极培育新型农业经营主体，新增家庭农场100家、农民合作社500家，培训新型农民20万人次。实施“互联网+”现代农业行动，打造创新型农业综合运营服务平台。三是强化科技化带动。严守耕地红线，藏粮于地、藏粮于技，推进粮食高产创建、绿色增产。以国家农业科技园区和8个省级农业科技园区为载体，每个县(市、区)建设一处高标准示范园区，两年内新建改建高效集约蔬菜温室10万个。大力实施“农业科技展翅行动”，力争引进新品种、新技术20个。四是强化品牌化带动。制定全市主要农产品生产规范和质量标准体系，新认证“三品一标”50个、基地20万亩，新增全国绿色食品原料生产基地1处，力争6个县(市、区)达到省级农产品质量安全县标准。持续抓好品牌建设，重点培育特色产业品牌10个、产品品牌20个，进一步打响“聊·胜一筹”农产品品牌。

　　大力推进工业提档升级。坚持创新发展、转型升级、提质增效，推动工业向“双中高”迈进，确保规模以上工业增加值、利税增长6%以上。一是延伸产业链条。做好强链的文章，推进八大传统产业转型升级，实施新一轮高水平技术改造，注入高端要素，打造更具市场优势的特色产业。做好补链的文章，抓好铜及铜加工、铝及铝加工、新能源汽车、化工新材料等产业链条前延后伸，补齐缺失环节，加快向终端、高端延伸。做好建链的文章，大力培育生物医药、节能环保、新能源、新材料等新兴产业，抓好冠晶蓝宝石、云龙电路芯片等10个重点项目，带动配套企业，建立产业链条，力争高新技术产业占比提高1.5个百分点。二是建设产业园区。突出抓好信发高新材料产业园建设，投资过亿元项目达到20个以上。加快祥光生态工业园、中通和时风新能源汽车产业园、东阿阿胶生物产业园、临清轴承产业园等重点园区建设，主要工业指标增幅高于全市2个百分点以上。积极培育开发区软件电子商务园、阳谷光电产业园等新兴产业园，培植新的经济增长点。三是培植产业集群。集中力量抓好汽车及机械装备等“八大产业集群”发展，做大做强骨干龙头企业，引进培育相关企业，做到纵向成链、横向成群、区域循环，打造特色优势产业集群。四是强化企业和项目支撑。大力实施“双百工程”，支持100家重点企业建立完善现代企业制度，向产业高端迈进;抓好中色奥博特金属粉体、新通新能源汽车配套产业园等100个工业重点项目建设，完成投资300亿元。

　　大力推进服务业扩量提质。推动服务业与先进制造业深度融合，服务业增加值占比提高1.5个百分点。一是大力发展文化旅游业。实施京杭运河聊城段保护开发，打造世界历史文化遗产、水利风景名胜、乡村特色旅游、温泉休闲度假目的地。实施东昌湖景区景观提升工程，重点培育古城观光游览、滨湖休闲度假、环湖健身娱乐等业态，努力建成全市综合性中央公园、国家5A级旅游风景区和世界文化景观遗产。启动阿胶小镇建设，推动阿胶景区创建国家5A级旅游景区。积极发展乡村旅游，培育旅游综合体22家，打造黄河文化旅游、水浒文化体验等8条精品线路。二是大力发展商贸物流业。着力建设大型城市综合体，抓好万达缤纷日月城、金田阳光城等10个重点项目建设，推动10家重点实体零售企业和10户大型专业市场创新转型。支持发展现代物流业，抓好20家重点企业发展，加快临清卓远能源交易中心、莘县亚世达冷链物流等5个重点项目建设。以创建国家电子商务示范城市为目标，加强聊城电子商务产业园建设和电商示范县创建，推广新凤祥“品牌+电商”模式，培育农村电商示范村25个。三是大力发展金融业。推动聊城农商银行股本结构调整，走出去设立分支机构。积极引进辖外金融机构，新引进金融机构4家。创新政金企对接和金融工作日制度，力争贷款余额增长10%。四是大力发展健康服务业。积极吸引社会资本投资医疗养老产业，加快市医养中心、和惠医养院和冠县新华康复医养院等10个重点项目建设，打造医养结合综合体。同时，抓好研发设计、商务咨询、家政服务、休闲娱乐、健身康复等其他服务业，推进服务业向高端化、多样化发展。

　　(二)坚定不移发展壮大新动能，切实增强发展内生动力。深入实施大项目带动、创新驱动、质量强市三大战略，为产业转型升级提供内生动力。一是着力增强优质高效投入的拉动力。突出抓好阿胶全产业链产业园等100个重点项目建设，切实优化投资结构，确保先进制造业项目占比达到35%以上。实行重点项目领导帮包制度，加强督导调度，确保计划项目100%开工、计划投资100%完成、计划竣工项目100%竣工。抓好重大交通项目建设，加快莘南、青兰高速建设进度，力争京九高铁、郑济高铁、德单高速、东阿至东平黄河大桥开工建设，做好聊泰铁路、聊城机场前期工作。二是着力增强科技创新的驱动力。推动规模以上企业全面建立研发机构，力争新建国家级创新平台2家、博士后科研工作站4家、省级创新平台14家。鼓励骨干企业牵头成立技术创新联盟，建设大型仪器设备协作共享平台、知识产权综合服务平台。深化产学研用协同创新，建成浙江大学聊城技术转移中心。统筹推进智慧城市“13510”工程，建设运行平台，促进大数据开发应用。三是着力增强质量品牌的竞争力。充分发挥五方机制的作用，加快创建全国质量强市示范城市。强化企业质量管理，推动大中型企业全面落实“首席质量官”制度，加强质量管理培训，开展“水城工匠”评选活动。强化标准引领，完善各领域标准体系，参与制修订国家标准、行业标准和省地方标准5项以上。强化平台支撑，加强鲁西质检中心建设，新扩建检验检测技术服务平台3个。强化品牌创建，着力培育东阿阿胶、魏氏熏鸡、济美酱菜等一批老字号品牌，中通、时风、泉林、金号等一批工业品牌，冠县鸭梨、莘县香瓜、茌平大枣、凤祥食品、丁马甲鱼等一批特色农产品品牌，乖宝、冠洲、三和等一批出口品牌，力争新增中国驰名商标2件、山东著名商标20件、山东名牌10个。四是着力增强人才队伍的支撑力。实施企业家培育“123”计划，加强高端对标培训，到2020年，培育10名左右在国内外具有较高知名度的优秀企业家，200名左右能带领企业在国内同行业处于领先地位的骨干企业家，3000名左右善于经营管理、具有良好发展潜力的成长型企业家;广泛开展聊城大讲堂活动，抓好对广大企业经营者的培训。加强科技人才队伍建设，组织开展多批次、常态化外国专家交流洽谈活动，举办人才招聘会50场以上，积极引进海内外高层次人才和团队;深化与聊城大学的合作，共同引进人才、培养人才。支持职业教育发展，鼓励建设特色名校、特色技师工作站，新增高技能人才5500人以上。各位代表，适应新常态，壮大新动能，创新是第一动力，人才是第一资源。我们一定要厚植良好生态、打造优质平台、营造浓厚氛围，切实做到广纳人才、留住人才、人尽其才，汇聚起聊城创新发展、转型升级的磅礴之力!

　　(三)坚定不移深化改革开放，不断激发经济发展活力。深化经济领域重点改革。一是积极推进农村综合改革。稳妥推进农村集体产权制度改革，探索农村土地征收、集体经营性建设用地入市、宅基地制度等项改革，赋予农民更多财产权。抓好新型农村合作金融试点，做好农村承包土地经营权抵押贷款试点工作。完成国有林场改革，深化供销社综合改革。二是深入推进企业改革。积极推动“个转企、小升规、规改股、股上市”，全市规模以上企业改制面达到10%以上，实现10家企业申报上市材料、10家企业在“新三板”挂牌，力争直接融资100亿元以上。深化国有企业改革，推进市属经营性国有资产集中统一监管，稳妥发展混合所有制经济，支持设立财务公司。三是加快推进投融资体制改革。加强财金、财信、担保、土储4个市级融资平台建设，加大国有资产整合注入力度，运用PPP、债券、专项建设基金等方式，促进融资结构多元化。设立产业转型、科技创新、城乡建设等六个方面的基金，总规模达到80亿元以上。四是大力推进民营经济发展。加大对“大众创业、万众创新”的支持力度，切实优化发展环境，新增省级创新创业孵化平台2家、小企业创业辅导基地3家，全市私营企业和个体工商户新增4万户。推进民营企业改革，鼓励进行资本多元化、社会化改造，参与国有企业改制。支持民营企业技术创新，培育省级“一企一技术”研发中心和创新企业20家。推动中小企业集群发展，培育营业收入过50亿元的产业集群18个，其中过100亿元的12个。

　　提升双向开放水平。一是在“融入”上聚焦。深度融入京津冀协同发展，主动承接北京非首都功能疏解和京津产业转移，加强在人才引进、科技成果转化、农产品供应等重点领域的协作，引进项目资金100亿元以上。积极融入中原经济区，加强与中西部城市的经贸往来，推进合作共赢、共同发展。更加主动地融入“一圈一带”，重点加强与济南的融合对接，主动承接济南辐射。二是在“招引”上发力。大力开展精准招商，精准选择重点区域、重点企业、重点项目，提高招商引资的针对性和实效性;拓宽招商引资渠道，加强与行业领军企业的对接，深化与友好城市、港澳台和各地商会协会的合作，确保每个省级工业园区至少落地2个投资过10亿元项目，全市到位资金800亿元以上，力争在引进世界500强、国内500强、大型央企和上市公司上取得突破。积极推进经济开发区转型升级，着力提高产业集聚效应和竞争力，确保主要经济指标占全市比重逐年提高。三是在“开拓”上突破。大力开拓国际市场，重点扩大新能源汽车、彩涂板、铜管件等高附加值产品出口，新建国家级出口质量安全示范区2个，新增有出口业绩企业50家。开拓先进技术设备和关键零部件进口渠道，促进企业技术改造。积极拓展海外投资，鼓励信发、祥光等企业扩大铝矾土、铜精矿等资源开发利用，支持乖宝宠物食品美国泰国投资、百佳食品海外仓储等项目建设。深化区域通关一体化改革，实行“月解读、季培训”制度，切实提升外经贸服务水平。

　　(四)坚定不移推进城乡协调发展，加快推进新型城镇化。抓住用好“一列入、三试点”的机遇，全面加强城乡建设，实现8.7万农村转移人口市民化和6万城中村、城边村原有居民市民化，常住和户籍人口城镇化率分别达到50%和40%。一是实施规划引领工程。坚持规划先行、多规合一、依规建设，着力提高规划水平和规划覆盖率。完成“聊茌东”都市区发展规划，推进茌平撤县设区，适时启动东阿撤县设区。全面完成县(市)城镇化发展规划，加快推进乡改镇、村改居。加快水系连通、综合交通等专项规划修编，做好高铁站区域及重点地段规划设计。二是实施功能提升工程。启动徒骇河、马颊河、京杭运河等水系连通工程，加快南城新湖项目建设，提升金牛湖、洛神湖、鱼丘湖和双海湖生态景观。完成海绵城市建设18平方公里，开工建设地下综合管廊12公里、建成6公里。中心城区新增供暖面积100万平方米;抓好月季园、凤凰苑和一批街心公园建设改造，新增绿化面积164公顷。三是实施道路畅通工程。完成黑龙江路东延和茌东大道建设。加快中心城区道路提升改造，抓好湖南路、西关街、财干路西延和花园路、卫育路改造提升等11个重点项目建设。实施治乱疏堵行动计划，打通道路微循环，依法治理乱停乱放、占道经营，坚决取缔非法营运三轮车，切实改善交通秩序、提高通行效率。四是实施精细管理工程。按照政企分开、管养分开原则，加快城管市场化改革，吸引社会资本投资参与城市管理。加强城镇环境和道路管理，对四个市辖区和七个县(市)实施分类考核。开展城市违法建设治理行动，不折不扣完成拆违任务。五是实施美丽乡村工程。对27个小城镇实施改造提升，建设完善新型社区20个。加强路域环境综合整治，推进农村河塘、沟渠连通治理，加快农村电网改造升级，力争完成农村无害化厕所改造，建设市级美丽乡村标准化示范片区38个。开展农村“户户通”道路硬化试点工作，整治村庄1500个，力争用三年时间基本实现全覆盖。

　　(五)坚定不移推进生态文明建设，努力营造良好人居环境。坚持以改善环境质量为核心，大力推进“三城共建”。一是坚决抓好过剩产能淘汰。严格执行安全、环保、能耗、质量、标准等法律法规，倒逼过剩产能退出。严厉打击制售“地条钢”等违法违规行为，关停一批小火电机组。二是坚决抓好能源节约。实施“工业绿动力”计划，开展重点用能单位“百千万”行动，万元生产总值能耗降低4.5%。抓好建筑节能，全面执行居住建筑75%、公共建筑65%的节能标准，装配式建筑占新建建筑比例达到10%左右。积极调整能源消费结构，开展太阳能应用示范市(县)建设;抓好茌平山东省农村地区供暖试点县工作，推进农村冬季清洁取暖。三是坚决抓好大气污染防治。强化燃煤污染防治，严格落实机组(锅炉)超低排放标准，普遍实施民用散煤综合治理，完成“以气代煤、以电代煤”工程10万户，确保7家清洁煤配送中心建成运营，压减燃煤127万吨;实施“气化聊城”工程，新增30个乡镇通上管道天然气，用三年时间实现“镇镇通”。强化工业大气污染治理，全面推行清洁生产，实施特别排放限值，加强挥发性有机物综合整治。强化城市面源污染治理，深入抓好扬尘整治和渣土运输管理，加快裸露土地绿化;持续开展加油站清理整顿和油品质量专项检查，严格高排放车辆监管。强化重污染天气应对，实施工业企业采暖季错峰生产，全面落实禁燃限放要求，做好气象、环境监测和预报预警。四是坚决抓好水污染防治。开展徒骇河、马颊河等主要河流沿线综合整治，落实“河长制”，确保河流水质达到地表水V类标准。推进工业企业污水处理设施和城镇污水处理厂提标改造，抓好信发电厂空冷机组改造和污水处理厂建设。加强村镇污水处理设施建设，70%建制镇、所有农村新型社区污水处理设施建成运营。全面完成“千亩表流、百亩潜流”人工湿地项目，全市湿地保护率达到66.8%。五是坚决抓好生态修复。划定并严守生态保护红线，严格落实“土十条”，开展重污染土壤治理和修复试点。做好国家森林城市创建工作，实施森林进城围城、村镇绿化美化、森林质量提升等六大工程，加快8个国有林场改造提升，完成造林面积20万亩。六是坚决抓好责任落实。严格考核奖惩，实行月通报制度，加大对各县(市、区)生态补偿力度。坚持环境保护“一岗双责”，加强网格化环境监管体系建设，对环保问题严重的地区和单位实施约谈、问责和“一票否决”。各位代表，碧水蓝天就是金山银山。我们一定要以壮士断腕的决心、背水一战的勇气，重拳治理环境污染，也期望各位人大代表、政协委员加强宣传引导，各位企业家主动承担社会责任，广大群众自觉参与环境保护，全市上下齐心协力，共同建设好我们的美丽家园!

　　(六)坚定不移保障改善民生，全面提高群众生活水平。坚决打赢脱贫攻坚战。加强革命老区建设，抓好各项扶贫政策落实，全面完成213个贫困村退出、8万贫困人口脱贫的目标，基本完成脱贫攻坚任务。一是大力推进产业扶贫。支持发展肉驴、畜禽、水产等特色养殖业，以及大棚蔬菜、材果兼用核桃、牡丹、元宝枫等特色种植业，培育一批旅游产业村，确保每个贫困村都有特色产业、每个贫困户都有增收项目。二是大力推进金融扶贫。建立扶贫开发基金，创新开发金融产品，发挥保险保障功能，形成政府融资平台为主导、社会各界参与的金融扶贫格局。三是大力推进企业扶贫。深入推进千企下乡，鼓励更多企业在贫困村建立生产基地，发展订单产业。四是大力推进基础设施扶贫。整体推进金堤河等四个重点片区集中连片开发，加快彭楼灌区改扩建工程建设，全面改善贫困村基础条件。五是大力推进教育医疗扶贫。完善贫困生资助体系，提升贫困村办学条件，对贫困家庭初高中毕业生开展订单、定岗、定向技能培训。深入推进“八个一”工程，推广“贫困户邻里互助护理”模式，对患病贫困人口实施分类救治。六是大力推进政策性兜底保障。实现农村低保与省定扶贫线“两线合一”，把丧失劳动能力的贫困人口全部纳入低保体系。各位代表，脱贫攻坚任务艰巨、使命光荣、群众期盼。我们一定要下定决心、排除万难，全力夺取脱贫攻坚新胜利，让全市人民一道奔向全面小康!

　　要扎实办好民生实事。把80%左右的财力用于民生领域，继续抓好10件实事。一是开展就业帮扶。统筹实施大学生“三支一扶”等基层服务项目，支持返乡下乡人员创业，发放创业担保贷款5亿元以上，免费开展技能培训3万人以上，扶持带动就业1.5万人以上。二是社会保险扩面提效。全面实施机关事业单位养老保险，完善职工大病保险、职工长期护理保险和居民大病保险。启动发行城镇职工社会保障卡，实现医疗保险市级统筹，推进跨省联网结算。三是建设中小学和幼儿园。加快推进“全面改薄”，建设校舍9.2万平方米。新建中小学32所、改扩建16所，建设校舍52万平方米，新增教学班1432个、学位数6.8万个，确保聊城一中新校、市特殊教育学校建成招生，基本解决城镇普通中小学“大班额”问题。新建改建公办幼儿园138所，支持社会力量兴办普惠性幼儿园。四是扩大实施卫生计生免费工程。在去年“五项免费”工程基础上，新增叶酸发放、孕前优生健康检查、白内障手术等三项免费项目。五是扶老救困。新增养老床位3600张。整合农村五保供养、城市“三无”人员救助制度，统筹实施城乡特困人员救助供养。六是改善居民居住条件。棚户区改造新开工3.5万套，基本建成2万套;改造老旧小区119个;完成存量贫困家庭危房改造。七是加强公共交通建设。新增新能源公交车230辆，优化公交线路，增加通行班次;完善城市公共自行车网络;中心城区新增停车位2000个。八是改善农村通行条件。新改建农村公路2500公里，改造农村危桥100座。九是实施文化惠民工程。建设村综合文化服务中心1000个，覆盖80%的行政村，省定贫困村基本实现全覆盖。完成文化惠民演出2000场，农村公益电影放映6万场，水城明珠剧场举办高水平演出50场。推进农村有线电视户户通，基本实现广电无线数字全覆盖。十是强化食品药品安全监管。加快“智慧监管”工程建设，推动莘县、东阿、茌平创建省级食品安全先进县，食品药品评价性抽检合格率达到96%以上。

　　积极推进社会事业发展和社会治理创新。实施基础教育综合改革、名校办分校、民办教育提升等五大工程，力争冠县、阳谷通过义务教育均衡县国家验收，东昌府、莘县通过省级评估。深入实施全民健康工程，推进公立医院法人治理结构改革，加强区域医疗联合体和基层医疗卫生机构标准化建设，扩大分级诊疗试点;开展出生人口性别比综合治理，增加产科、儿科医护人员和床位设施，市县两级均建成危重孕产妇和危重新生儿救治中心。大力发展文化事业，加快“一校三馆”、县级国家综合档案馆和档案信息化建设，继续抓好二轮修志。全面发展群众体育、竞技体育，举办第七届全民健身运动会。深化平安聊城建设，开展基层基础建设三年攻坚战，建立“一村一警务助理”制度，完善立体化社会治安防控体系。狠抓安全生产责任体系落实，加强重点领域安全监管和隐患治理，推进乡镇消防队伍建设，坚决遏制重特大事故发生。加快社会信用体系建设，建立市级信用信息交换共享平台。做好双拥共建工作，推动军民深度融合发展。扎实做好社会科学、民族宗教、妇女儿童、气象地震、残疾人、老龄、人防、油区、对口支援等工作，确保社会和谐稳定。

　　(七)坚定不移加强自身建设，持续提升政府履职能力。站在新起点、面对新形势、实现新目标，各级政府责任重大。我们要牢固树立政治意识、大局意识、核心意识、看齐意识，深入开展“两学一做”学习教育，进一步加强法治、创新、责任、服务和廉洁政府建设。

　　不忘初心，践行为民之本。要坚定不移站稳人民立场，把政策向民生聚焦、资源向民生倾斜、服务向民生覆盖，扎实为群众办实事、解难题。以敬民之心，行简政之道，深化“放管服”改革，推进行政审批、商事制度、中介收费等重点领域改革，市县两级行政许可事项全部纳入网上运行;加强事中事后监管，实现“双随机、一公开”制度全覆盖。深入落实“企业工作日”、“金融工作日”、“群众工作日”制度，推进政务公开，开展政风行风民主评议，畅通群众来信来访、建议征集、市长公开电话等渠道，及时解决企业和群众反映的困难和问题。

　　担当有为，大兴实干之风。更加突出问题导向，深入推进政风转变，做到勇于作为、敢于担当。聚焦动力不足“不想为”的问题，强化正向激励，旗帜鲜明地为担当者担当，切实保护和调动干部队伍干事创业的积极性;建立健全问责问效机制，严厉惩处失职渎职行为。聚焦担当不足“不敢为”的问题，加快构建容错纠错机制，消除干部队伍的后顾之忧。聚焦能力不足“不会为”的问题，加强教育培训、交流锻炼，努力克服本领恐慌。聚焦效率低下“慢作为”的问题，加强目标责任清单管理，建立健全月调度季通报、视频内参、电视问政等制度，强化考核奖惩和结果运用，促进各项工作提质增效。

　　依法而行，恪守施政之规。按照授予有据、行使有规、监督有效的原则，切实规范行政行为。积极配合人大立法，及时提出立法需求和建议。自觉接受人大及其常委会的法律监督、工作监督和政协的民主监督，虚心接受社会监督和舆论监督。认真落实重大行政决策法定程序，主动征求人大代表、政协委员和重大决策咨询委员会、法律顾问等方面的意见建议，做好人大代表议案建议和政协委员提案办理工作。推进综合行政执法体制改革，加强行政复议规范化建设，进一步做好行政应诉工作。完善行政调解和行政裁决制度，促进仲裁工作健康发展。加强和改进政府信访工作，依法化解矛盾纠纷和历史积案，切实维护公民、法人和其他组织的合法权益。

　　从严治政，彰显清廉之道。坚持“一岗双责”，严格落实全面从严治党政治责任，深入推进廉政建设和作风建设。加强理想信念和道德品质教育，正本清源、固根守魂。坚持开正门、堵旁门、关后门，减少自由裁量权，从源头上防止设租寻租和利益输送。完善国库集中支付制度，推进预算绩效管理和“三公”经费公开，一般公用经费支出压减5%。严格规范招投标制度，建立市县统一规范的交易平台，提高政府采购效率。推进公共资金、国有资产资源和领导干部履行经济责任情况审计全覆盖，加强审计结果运用。严格落实中央八项规定精神和省委、市委实施办法，坚决防止“四风”反弹回潮。坚持有腐必反、有贪必肃，加大违纪违法案件查处力度，努力打造绝对忠诚、干事担当、干净自律、充满活力的干部队伍。

　　各位代表，同心共筑小康梦，携手谱写新篇章。让我们更加紧密地团结在以习近平同志为核心的党中央周围，在省委、省政府和市委的坚强领导下，凝心聚力，尽责担当，奋发进取，撸起袖子加油干，为全面建成小康社会、创造聊城人民更加美好的幸福生活而努力奋斗，以优异成绩迎接党的十九大胜利召开!
